# HIV Infection, Genital Symptoms and Sexual Risk Behavior among Indian Truck Drivers from a Large Transportation Company in South India

**DOI:** 10.4103/0974-777X.52977

**Published:** 2009

**Authors:** Annie Dude, Ganesh Oruganti, Vinod Kumar, Kenneth H Mayer, Vijay Yeldandi, John A Schneider

**Affiliations:** 1 *Pritzker School of Medicine, University of Chicago, Chicago*; 2 *International Center for Human Health Advancement, SHARE – India, Hyderabad*; 3 *Chest Hospital, Hyderabad, Andhra Pradesh*; 4 *The Miriam Hospital and Brown University School of Medicine*; 5 *Departments of Medicine and Health Studies, University of Chicago, Chicago*

**Keywords:** HIV, India, Truck driver

## Abstract

**Background:**

Sentinel surveillance conducted in the high Human Immuno-deficiency Virus (HIV) prevalent state of Andhra Pradesh includes sub-populations thought to be at high-risk for HIV, but has not included truck drivers. Novel HIV prevention programs targeting this population increasingly adopt public - private partnership models. There have been no targeted studies of HIV prevalence and risk behavior among truck drivers belonging to the private sector in India.

**Methods:**

A sample of 189 truck drivers, aged between 15 and 56, were recruited from Gati Limited's large trucking depot in Hyderabad, India. A quantitative survey instrument was conducted along with blood collection for HIV 1/2 testing. Multivariate regression models were utilized to determine predictors of HIV infection and risk behavior.

**Results:**

2.1% of subjects were infected with HIV. Older age was protective against self-reported genital symptoms (OR = 0.77; *P* = 0.03), but these were more likely among those truck drivers with greater income (OR = 1.05; *P* = 0.02), and those who spent more time away from home (OR = 25.7; *P* = 0.001). Men with higher incomes also reported significantly more sex partners (OLS coefficient = 0.016 more partners / 100 rupees in monthly income, *P* = 0.04), as did men who spent a great deal of time away from home (OLS coefficient = 1.30, *P* = 0.002). Drivers were more likely to report condom use with regular partners if they had ever visited a female sex worker (OR = 6.26; *P* = 0.002), but married drivers exhibited decreased use of condoms with regular partners (OR = 0.14, *P* = 0.008). Men who had higher levels of knowledge regarding HIV and HIV preventative practices were also more likely to use condoms with regular partners (OR = 1.22, *P* = 0.03).

**Conclusion:**

Time away from home, urban residence, income, and marital status were the strongest correlates of genital symptoms for Sexually Transmitted Infections (STI) and risk behaviors, although none were consistent predictors of all outcomes. Low HIV prevalence might be explained by a cohort that was mostly married, and at home. Novel HIV prevention interventions may be most cost effective when focusing upon young, single, and long-haul truck drivers.

## INTRODUCTION

South/Southeast Asia is the second-most HIV affected region in the world, and India continues to have the highest numbers of HIV-infected individuals in Asia.[1] The Indian state of Andhra Pradesh (AP) can be described as a country within a country. Andhra Pradesh has its own distinct language, (Telugu) and with a population of 83 million, is larger than countries like Vietnam or Germany. It has the highest rates of HIV infection in the country due to sexual transmission.[2,3] Located in the center of the country, the capital of AP, Hyderabad, is directly connected to five other major cities and high-HIV prevalence districts in coastal AP through national and state highway systems.

Sentinel surveillance conducted by the state of Andhra Pradesh has included most sub-populations thought to be athigh-risk for HIV and STIs, but has not included truck drivers.[4] Despite this gap, truck drivers are consistently thought to be at increased risk for HIV and other STIs,[5] some of whom are targeted by state and private programs.[6] With three million trucks, often with both a driver and younger male helper or cleaner, on the roads in India,[7] there are surprisingly few research studies examining HIV or STI prevalence in truck drivers in India, and all have been cross-sectional in nature.[8–11] These have taken place at generally high – risk roadside stops or border areas where sex workers are easily available. In one large study, truck drivers were sampled from trans-shipment locations; however, residence information was absent and short distance truck drivers were excluded.[8] The sustainability of programs currently under evaluation such as the Bill and Melinda Gates Avahan program are important.[12] However, it has become increasingly clear that public - private partnerships, and in particular the private sector, will be needed to maintain pressure on behavioral change and education and motivation programs for high – risk mobile populations, such as truck drivers.[13,14]

This study aims to understand the extent of risky sexual behaviors among a group of truck drivers working within a large transportation company based in Andhra Pradesh, India, a state with the highest rates of HIV infection due to sexual transmission. We examine the sociodemographic and work-related predictors of risky sexual behavior, HIV, and genital symptoms of a sexually transmitted infection.

## MATERIALS AND METHODS

### Quantitative survey development

We based the survey instrument on HIV risk factor surveys used by our group previously;[15] we primarily assessed HIV risk taking behavior and hygiene practices. For the majority of behavioral outcome items, we included the responses “yes”, “no,” and “unknown” to account for cross-cultural differences in subjective responses to larger Likert type scales.[16] The survey covered areas including sociodemographics, sexual behavior and partnering patterns, HIV and STI transmission knowledge, previous STI diagnoses, and genital symptoms. We initially developed the quantitative survey instrument in English, then translated it into both Hindi and Telugu, the major languages spoken by study subjects in Andhra Pradesh. Counselors familiar with all three languages, the local culture, and the approximate health literacy of the study subjects, all helped to develop the initial English surveys as well as undertook translation into Hindi and Telugu.

### Study subject recruitment and survey administration

The truck drivers we recruited into the study were at least 18 years of age, fluent Hindi or Telugu speakers, and employed or contracted by Gati Limited, one of the largest transport companies in India.[17] Study staff recruited truck drivers directly from Gati's main depot center in Bowenpally, just outside of Hyderabad, the capital of Andhra Pradesh.

Interested participants were led to a private room in the clinic where informed consent was obtained, followed by the survey. We tested all participants for HIV-1 and HIV-2 using three sequential ELISAs (Genscreen HIV 1/2, Bio-Rad; Detect HIV, Adaltis; HIV Tri-Dot, Biotech Inc.) in accordance with the national guidelines. Gati staff were not involved in the recruitment process and did not have access to the study data. The Institutional Review Boards at the MediCiti Hospital, the Miriam Hospital, and the University of Chicago approved all final protocols.

### Data analysis

The final analytic sample included all recruited males who reported having had sex at least once, and who had complete data on all outcome and control variables, with a final N = 189. All data were analyzed using Stata Release 8.0 (StataSoft Corp, Austin TX). We employed logistic regression to estimate the predictors of HIV infection, any reported genital symptoms in the past 12 months, whether a respondent reported ever visiting a female sex worker, whether he reported visiting a female sex worker in the past six months, and whether he reported using a condom with his regular partner during the last sex. Finally, we used Ordinary Least Squares (OLS) regression to examine correlates of how many sexual partners a respondent had during the prior 12 months. *T-tests* on the coefficients obtained from the regressions were utilized to determine the statistical significance of each covariate; covariates were considered significant if the *P* -value associated with the t statistic of the coefficient was <0.05.

In order to control for the level of knowledge an individual held regarding HIV, HIV prevention, and HIV transmission in our final models, we constructed a count variable tallying the number of correct responses that respondents gave to a battery of nine questions. We also enquired whether a respondent reported having heard of HIV, as well as whether he reported being ‘aware’ of other sexually transmitted infections, for a total of 11 questions in the HIV knowledge count variable. Our regression models also controlled for a respondent's age, monthly income, marital status, number of children, urban residence, educational attainment, time spent away from home, alcohol use, and other sexual behaviors.

## RESULTS

### Summary statistics

Summary statistics for the analytical sample are found in Table 1. Most drivers had spent less than six months away from home in the past year, although 11.1% spent more than six of the past 12 months away from home. While 29.1% of the total sample reported ever visiting a female sex worker, only 11.6% of the total reported a visit to a female sex worker in the past six months; 19.1% of the total respondents indicated that they used a condom during their last sex with their regular partner, although this figure masks considerable heterogeneity in condom use according to marital status, as 75.0% of unmarried men report condom use with their last regular partner, compared to only 10.9% of married men. In this sample, 2.1% tested positive for HIV, 3.2% self-reported a previous STI diagnosis, and 8.5% reported experiencing genital STI symptoms (burning upon urination, genital discharge, or genital ulcers/sores) in the past 12 months.

**Table 1:** Sample summary statistics (N = 189)

Variable	Mean	Standard deviation
Demographic characteristics		
Current age (years)	31.2	61.4
Number of children	1.5	1.4
Income (100s of rupees/month)	36.8	15.6
Sexual behavior		
Number of sex partners reported from past 12 months	1.5	2.1
**Demographic characteristics**	**Percentage**	
Currently married	87.3	
Residential location		
From urban/suburban area	50.8	
From village/rural area	49.2	
Education		
Incomplete primary or none	14.3	
Complete primary	16.4	
Complete secondary	62.4	
Some college/college graduate	6.9	
Time spent away from home in past year		
6 months or less	88.9	
> 6 months	11.1	
Sexual behavior		
Has ever visited a female sex worker (fsw)	29.1	
Has visited a fsw in the past six months	11.6	
Always uses condoms with sex workers in the past six months	59.1 1*	
Used a condom at last sex with regular partner		
All men	19.1	
Married men	10.9	
Unmarried men	75.0	
Has engaged in sex with a male partner in the past six months	0.5	
Alcohol use		
Daily/more than twice weekly	43.4	
Medical outcomes		
HIV positive	2.1	
Reports previous STI diagnosis	3.2	
Any genital symptoms in the past 12 months	8.5	

* N for this percentage equals 22

### HIV transmission and prevention knowledge

Table 2 indicates that, while 87.8% of the sample report having heard of HIV and 75.7% are aware of sexually transmitted infections, these men hold considerably less accurate knowledge concerning HIV transmission, prevention, and treatment. In particular, only 60.3% indicate that using a condom correctly can help prevent HIV, and only slightly more than half (54%) report that reducing visits to commercial sex workers or remaining monogamous can protect against infection; 37.0% of the sample believe that HIV can be cured, and only 52.9% of these men realize that a healthy-looking person can contract HIV. 60.9 and 62.4%, respectively, believe that HIV can be transmitted by sharing food or shaking hands with someone with HIV, yet only 50.3 and 65.1% correctly identify blood transfusions and sharing intravenous injection equipment as potential sources of infection.

**Table 2:** HIV awareness and knowledge (N = 189)

Question	Correct answer	% reporting correct answer
Have you heard of HIV or AIDS?	Yes	87.8
Are you aware that there are some diseases that can be passed through sexual intercourse?	Yes	75.7
Can the correct use of a condom every time a person is engaged insexual intercourse help protect him/her from the HIV virus?	Yes	60.3
Can a person contract HIV through the sharing of food with an infected person?	No	60.9
Can a person contract HIV by shaking the hand of an infected person?	No	62.4
Can a person contract HIV through injections with a needle previously used by someone else?	Yes	65.1
Can a person contract HIV from an infected blood source i.e. transfusion?	Yes	50.3
Can a person protect himself/herself from HIV by abstaining from sexual intercourse especially with multiple partners or female sex workers?	Yes	54.0
Can a healthy appearing person be infected with HIV?	Yes	52.9
Based on your current understanding of HIV, is there medication that can cure HIV?	No	63.0
Can the HIV infection in a pregnant woman be passed on to her unborn child?	Yes	63.0

Individuals who responded ‘unsure’ or ‘don't know’ were categorized as giving an incorrect answer

### Medical outcomes

Table 3 presents predictors of HIV infection and genital symptoms from multivariate regression models. Married truck drivers were 0.03 times as likely as unmarried truck drivers to have a positive HIV test (*P* = 0.06). Truck drivers who spent more than six months away from home were much more likely to report genital STI symptoms than those who spent less time away (OR = 25.74; *P* = 0.001). The risk of reporting STI symptoms increased with increasing income: drivers were 1.05 times as likely to report symptoms for each additional 100 rupees of income they earn per month (*P* = 0.02). Finally, increased age was negatively associated with STI symptoms (OR = 0.77, *P* = 0.03). Risky sexual behaviors such as recent visits to female sex workers (FSWs) and more sexual partners, were not significantly associated with either HIV or STI symptoms.

**Table 3:** Predictors of HIV infection and STI genital symptoms

Variable	HIV (N = 189)			Genital symptoms (N = 189)		
		
	Odds ratio	95% confidence interval	*P*-value	Odds ratio	95% confidence interval	*P*-value
Demographic characteristics						
Current age (years)	0.98	0.75 - 1.27	0.87	0.77	0.61 - 0.97	0.03
Income (100s of rupees/month)	1.01	0.92 - 1.11	0.85	1.05	1.01 - 1.10	0.02
Currently married	0.03	0.00 - 1.20	0.06	0.41	0.02 - 7.17	0.55
Number of children	2.58	0.90 - 7.45	0.08	1.27	0.50 - 3.22	0.62
Residential location						
Urban residence	0.24	0.01 - 5.63	0.38	5.90	0.91 - 38.1	0.06
Education						
Education (ordinal) (r = incomplete primary)	4.26	0.55 - 32.36	0.16	1.61	0.64 - 4.03	0.31
Time spent away from home in the past year						
> 6 months away from home (r = 6 months or less away)	n/a2			25.74	4.06 - 163.06	0.00′
Sexual behavior						
Has ever visited a female sex worker (fsw)	4.73	0.15 - 145.3	0.37	1.96	0.32 - 11.92	0.46
Has visited a fsw in the past six months	n/a2			0.74	0.03 - 16.27	0.85
Used condom at last sex with regular partner	4.67	0.19 - 116.1	0.52	0.90	0.09 - 8.69	0.93
Number of sexual partners in the past twelve months	0.42	0.03 - 5.86	0.35	1.33	0.77 - 2.27	0.30
Alcohol use						
Frequent alcohol use (r = weekly or no alcohol use)	0.83	0.07 - 9.38	0.88	1.03	0.21 - 4.98	0.97
HIV knowledge						
HIV knowledge count	0.79	0.55 - 1.15	0.22	1.01	0.78 - 1.33	0.92

*P*-value obtained from *t*-tests determining whether reported odds ratios are significantly different from 1 (two-tailed test); Model fails to converge when this covariate is included

### Sexual risk behaviors

Ordinary Least Squares regression coefficients predicting the number of sexual partners a respondent reports having had in the past 12 months are presented in Table 4. Not surprisingly, individuals who report having visited FSWs in the past six months report significantly more partners; almost 3.5 more partners than those who have not frequented FSWs in the recent past (*P* < 0.001). More time spent away from home was strongly correlated with reporting more partners, with a respondent reporting, on average, 1.30 more partners if he spent more than six months of the past year away from home (*P* = 0.002). Increased income was also associated with increased number of partners (0.016 additional partners for each additional 100 rupees in monthly salary; *P* = 0.04).

**Table 4:** Predictors of the number of sex partners reported over the past 12 months

Variable	All men (N = 189)			Married men (N = 165)			Unmarried men (N = 24)		
			
	OLS Coefficient	95% confidence interval	*P*-value1	OLS Coefficient	95% confidence interval	*P*-value1	OLS Coefficient	95% confidence interval	*P*-value
Demographic characteristics									
Current age (years)	0.001	-0.045-0.048	0.96	-0.004	-0.044-0.036	0.84	0.165	-0.230-0.560	0.39
Income (100s of rupees/month)	0.016	0.0005-0.032	0.04	0.007	-0.007-0.021	0.34	0.102	-0.014-0.219	0.08
Currently married	0.032	-0.843-0.908	0.94	n/a	n/a	n/a	n/a	n/a	n/a
Number of children	-0.136	-0.353-0.082	0.22	-0.128	-0.312-0.057	0.17	n/a	n/a	n/a
Residential location										
Urban residence	-0.005	-0.498-0.487	0.98	-0.009	-0.450-0.431	0.97	0.103	-3.671-3.876	0.95
Education										
Education (ordinal) (r = incomplete primary)	0.048	-0.247-0.343	0.75	-0.084	-0.341-0.173	0.52	2.018	-0.271-4.308	0.08
Time spent away from home in the past year									
>6 months away from home (r = 6 months or less away)	1.30	0.47-2.13	0.002	1.43	0.67-2.19	0.000	2.271	-2.456-6.998	0.32
Sexual behavior									
Has ever visited a female sex worker (fsw)	-0.008	-0.677-0.661	0.98	-0.155	-0.768-0.457	0.62	1.367	-2.035-4.770	0.40
Has visited a fsw in the past six months	3.52	2.58-4.45	0.000	3.87	2.88-4.87	0.00	4.431	-0.199-9.062	0.06
Alcohol use									
Frequent alcohol use (r = weekly or no alcohol use)	-0.418	-0.904-0.069	0.09	-0.133	-0.573-0.308	0.55	-2.24	-5.58-1.10	0.17
HIV knowledge									
HIV knowledge count	-0.005	-0.076-0.066	0.90	-0.012	-.077-0.052	0.70	-0.041	-0.390-0.308	0.81
Constant	0.650	-0.93-2.24	0.42	1.342	0.048-2.64	0.04	-10.24	-21.20-0.71	0.07

*P*-value obtained from t-tests determining whether reported odds ratios are significantly different from 1 (two-tailed test), OLS = Ordinary least squares

Determinants of the number of sexual partners differed according to marital status. For married men, time spent away from home, as well as visits to FSWs, were significant correlates of greater numbers of sexual partners, with married men reporting 1.43 additional sexual partners if they also spent more than six months away from home (*P* < 0.001) and 3.87 additional sexual partners in total if they visited an FSW in the past six months (*P* < 0.001). For unmarried men, neither time away from home nor visits to FSWs are significantly associated with a greater number of sexual partners.

Table 5 presents correlates whether the respondent used a condom at last sex with his regular partner. Marital status was a strong predictor of behavior. Currently married men were 0.14 times as likely to report condom use with their regular partners as currently unmarried men (*P* = 0.008). Those married men that did report condom use with regular partners (column 2) were much more likely to have visited an FSW, both ever (OR = 7.46; *P* = 0.004) and in the past six months (OR = 5.79; *P* = 0.09). These married men that use condoms were also more likely to exhibit nonsexual risk behavior: they were 3.01 times as likely to report ingesting alcohol more than twice a week than married men who do not use condoms with their wives (*P* = 0.07). Finally, among married men, each additional child resulted in a 0.56 decrease in the likelihood of using condoms (*P* = 0.04).

**Table 5:** Predictors of condom use at last sex with regular partner

Variable	All men (N = 189)			Married men (N = 165)		
		
	Odds ratio	95% confidence interval	*P*-value^1^	Odds ratio	95% confidence interval	*P*-value
Demographic characteristics						
Current age (years)	1.03	0.94 - 1.13	0.56	1.05	0.94 - 1.17	0.37
Income (100s of rupees/month)	1.01	0.98 - 1.04	0.63	1.01	0.97 - 1.04	0.73
Currently married	0.14	0.03 - 0.59	0.008	n/a	n/a	n/a
Number of children	0.64	0.38 - 1.06	0.09	0.56	0.32 - 0.98	0.04
Residential location						
Urban residence	0.52	0.18 - 1.48	0.22	0.56	0.17 - 1.86	0.35
Education						
Education (ordinal) (r = incomplete primary)	0.55	0.29 - 1.08	0.08	0.51	0.25 - 1.06	0.07
Time spent away from home in the past year						
> 6 months away from home (r = 6 months or less away)	0.38	0.06 - 2.31	0.29	0.21	0.02 - 2.24	0.20
Sexual behavior						
Has ever visited a female sex worker (fsw)	6.26	1.93 - 20.29	0.002	7.46	1.93 - 28.82	0.004
Has visited a fsw in the past 6 months	2.15	0.39 - 11.73	0.38	5.79	0.77 - 43.65	0.09
Number of sexual partners in the past twelve months	1.02	0.81 - 1.29	0.84	0.94	0.66 - 1.33	0.72
Alcohol use						
Frequent alcohol use (r = weekly or no alcohol use)	2.07	0.76 - 5.65	0.16	3.01	0.91 - 9.96	0.07
HIV knowledge						
HIV knowledge count	1.22	1.03 - 1.46	0.03	1.14	0.93 - 1.40	0.20

*P*-value obtained frotm *t*-tests determining whether reported odds ratios are significantly different from 1 (two-tailed test); Model fails to converge when this covariate is included

Visiting an FSW was significantly associated with using condoms in the larger sample (OR = 6.26; *P* = 0.002), although this association was not significant for visits to FSWs in the past six months (OR = 2.15; *P* = 0.38). Finally, increased knowledge of HIV transmission and prevention was significantly and positively associated with condom use in the large sample, as each correct answer to an HIV knowledge question increased the likelihood of using a condom at last sex with a regular partner by 1.22 (*P* = 0.03). This association, however, was not statistically significant in either the subsample of currently married men (OR = 1.14; *P* = 0.22) or the subsample of unmarried men (data not shown; OR = 1.65; *P* = 0.10). Increased educational attainment, however, as opposed to specific knowledge regarding HIV, was actually associated with less condom use, both in the larger sample (OR = 0.55; *P* = 0.08) and among married men (OR = 0.51; *P* = 0.07).

## CONCLUSION

These data from a large transportation company in a high HIV prevalence state of India generally support findings from previous studies.[8,9,18] Respondents with fewer social constraints on behavior, who spend more time away from home, and who are not yet married, were more likely to engage in risky behaviors such as having multiple sexual partners.

Furthermore, these data indicate that truck drivers from a large transportation company in Andhra Pradesh have an HIV prevalence rate approximately twice that of the general adult population of the state.[19] Yet, the HIV prevalence of 2.1% among these truckers is lower than that in other surveys of truck drivers in South Asia.[8,20] These results indicate that, while truckers in Andhra Pradesh still have higher rates of HIV than the general population, there is still space to prevent future HIV infections among this group, and from this group to the general state population. Culturally sensitive informational and motivational interventions can increase condom use among truck drivers with both their marital and nonmarital partners.[21]

However, patterns of risky behavior might prove resistant to change.[22] Our results confirm that many truck drivers behave in sexually risky ways: 11.6% of them report having visited a sex worker in the past six months, and 15.3% report having multiple sexual partners in the past year. The present data, however, also indicate lower levels of risk-taking behavior among truck drivers than in past surveys. Compared to previous studies which found that over 80% of drivers admitted to having sex with FSWs,[10,23] these truckers were much less likely to report this behavior, even when considering the high percentage of those who admit to having ever visited an FSW (29.1%). While these results could represent a real decline in the number of truckers visiting sex workers, they could also be due to the higher socioeconomic status of this sub-population of truck drivers, under-reporting, a greater number of short-haul truck drivers in the sample or potentially other unidentified data flaws, given higher rates of HIV infection reported most recently from coastal regions of the state.[8]

The results regarding condom use are less encouraging. While 19.1% of the subjects report using condoms with their regular partners, and 59.1% of those who report FSW visits in the past six months report condom use during these visits, these numbers are comparable to condom use rates found in surveys among truck drivers conducted five to ten years ago.[11,23,24] These relatively low rates of condom use persist despite the fact that most individuals in this survey are aware of HIV, and also report that they are aware that condoms can prevent HIV. Individuals who visit FSWs are much more likely to use condoms with both marital and nonmarital partners, and it seems there is some recognition of the utility of using condoms when engaging in certain behaviors and that at least some of this population is amenable to behavior change.

Married men were much less likely than nonmarried men to use condoms, especially married men with many children. Condom use within marriage, or with intimate partners could send a strong signal of infidelity, and thus both partners have a disincentive to insist on using them.[25] The historical use of sterilization as contraception in India might also preclude any further need or discussion within married couples regarding any method of contraception, whether for family planning or for sexually transmitted infection prevention.

These data indicate that risky activity is far from universal in this population. In particular, married and rural men are statistically significantly less likely to visit FSWs, indicating that all truck drivers are not equal in their risk behavior. Those whose schedules permit them to remain closer to home are also much less likely to engage in risky behaviors, and to report genital symptoms of an STI. Thus, while addressing risky sexual behavior among drivers could serve as a useful means of preventing infections from spreading to the general population, different groups of drivers might require different prevention messages tailored to the likelihood of engagement in risky behavior. Even among married men, there seems to exist a group that engages in more sexual and nonsexual risk behavior, and are open to behavior change, as these men are more likely to use condoms than other married men.

A lack of basic information regarding HIV, HIV transmission, and HIV prevention might also be a barrier to behavior change in this sample. While 87% individuals had heard of HIV, only between 50-75% could identify correct answers regarding different modes of viral transmission and ways of protecting oneself from HIV. These numbers are comparable to those reported in a population-based survey from Andhra Pradesh. In this study 93% of men reported having heard of HIV and 73% of the men knew that consistent condom use could protect against HIV.[26]

Overall, the results from this study indicate that there is a need to identify more completely the structure of the sexual and social networks of truck drivers, their wives, commercial sex workers, and other sexual partners and to develop effective bio-behavioral HIV prevention interventions in truck drivers. Furthermore, longitudinal research that enhances long-term behavior change, and explores the effectiveness of novel biological prevention interventions implemented in truck drivers, could give direction and strategies to reduce HIV and other STI risks in this highly mobile population.
